# The Ethnic and Geographical Distribution of Fabella: A Systematic Review and Meta-Analysis of 34,733 Knees

**DOI:** 10.7759/cureus.14743

**Published:** 2021-04-28

**Authors:** Adil Asghar, Shagufta Naaz, Binita Chaudhary

**Affiliations:** 1 Anatomy, All India Institute of Medical Sciences Patna, Patna, IND; 2 Anaesthesiology, All India Institute of Medical Sciences Patna, Patna, IND

**Keywords:** odds ratio, prevalence, knee joints, osteoarthritis, age-related degeneration

## Abstract

Introduction: The primary studies demonstrated that fabellar prevalence (FP) varied with ethnic and geographical distribution. Osteoarthritis (OA) and age-related degeneration have a significant association with FP. The prevalence of OA worldwide was doubled with life expectancy. Increased life expectancy has increased exposure to OA and age-related degeneration which could be a possible reason for the rise of FP. The analysis was conducted to provide insight about FP in respect to geographical, ethnic, sex, and laterality distribution.

Methodology: Eighty-six studies were included which have data from 34,733 knee joints. Fifty radiological studies were consisting of 27,293 knees and 36 cadaveric studies had the data of 7,440 knees of dissected specimens, respectively. The prevalence, Odds, and rate ratios were calculated for aging, osteoarthritis, and ethnic variation.

Results: The worldwide FP was 25% (95% CI, 0.22, 0.28). The prevalence of fabella was found to be higher in cadaveric studies (32%) than radiological studies (19%) with significant heterogeneity. The FP was 16-18% till 1950 which was doubled by 2020 (35%). The FP in OA knee was 51% which was thrice of baseline.

## Introduction

The tendon of the lateral head of gastrocnemius has a small fibrocartilaginous or ossified nodule. This nodule is a sesamoid bone named fabella which develops inside the tendon after 8-12 years of age [1]. The fabella is making posterolateral or fourth compartment of the knee and it is articulating with the lateral condyle of the femur at the posterior aspect. The fabella redirects the pull of the lateral head of gastrocnemius to improve the efficiency of this muscle. It prevents friction-induced damage to the tendon [2]. The fabella is present as a cartilaginous nodule that is ossified in the later phase of the first decade by enchondral ossification [3]. The ossified fabella is usually visible in routine radiographs of the knee. This normal anatomical variant is often confused with osteophytes or intra-meniscal calcifications or intra-articular loose bodies in degenerated knees with osteoarthritis (OA). The ossification of fabella is not only under genetic control but also under influence of local mechanical stress and biomechanical need which may lead to larger dimensions of the fabella [2]. The fabella is connected with the apex of the fibula by the fabellofibular ligament. The fabellofibular ligament is the thickening of the distal part of the biceps tendon (short head) and maintains the posterolateral stability of the knee joint. Its thickening is considered to be responsible for the discomfort in the posterolateral corner or compartment of the knee, known as fabella syndrome [4]. Earlier, the fabella is rarely affected by disorders, like chondromalacia, osteoarthritis, dislocation, and fracture which resulted in fabella syndrome or entrapment syndrome of common fibular nerve or popliteal artery [2,4]. The fabellar impingement with prostheses is common after total knee arthroplasty (TKA) and may cause knee disorders. Because of the growing number and expectation of TKA patients, clinicians are becoming more concerned about these issues [1,4].

The fabellar prevalence (FP) is variable, which depends upon the methodologies, and racial or geographical distributions. Berthaume et al. [5] claimed that the median prevalence of fabella has raised by 3.5-folds in the last 150 years that could be evolutionary to meet the biomechanical demands. Some researchers found higher FP in older age groups [6,7]. We aimed to measure the prevalence of fabella and its distribution worldwide in a larger sample size. The prevalence of fabella is studied in the different age groups and geographical distribution. The effect of gender, ethnicity, laterality along with osteoarthritis will also be studied in the larger sample size.

## Materials and methods

The inclusion criteria for this meta-analysis were the published articles, conference abstracts, unpublished studies with obtainable data, and electronic mails used to gather unpublished data from authors of published articles. The case series or literature review were included in the analysis if sample sizes were mentioned. Their references may be utilized to explain the findings. Both cadaveric and radiological studies were included. The data of radiographs, CT, and MRI scans were considered.

The mean prevalence mentioned in the textbooks or published literature without sample references was excluded. If the authors of any radiographic study were confused between popliteal artery calcification and fabella, then such data were excluded from the analysis. The data of USG and PET excluded from analysis due to poor sensitivity. The literature associated with mammalian prevalence other than human was excluded based on the titles, abstracts, and full articles. Any reports or case series were excluded if the sample size not mentioned. 

The systemic search was conducted in the primary database like Medline, Embase, Pubmed, Ebsco, Google Scholar, Ovid database, AUSPORT, and Cochrane library till June 2020. The print or online journals of anatomy, orthopedics, sports or biomechanics, morphology or anthropology science, radiology, and radiotherapy were thoroughly investigated. The search strategy consisted of MeSH terms in different strings of permutations or combinations with Boolean operators, e.g., prevalence, fabella, osteoarthritis, aging, and knee pain.

Selection of studies and assessment of the risk of bias

The studies collected from search strategies were shortlisted with the help of Reyyan QCRI app. The data of search strategies were imported to Reyyan QCRI and shortlisted based on inclusion and exclusion criteria and reading the abstract. A total of eighty-six studies were found suitable for further assessment (Table 1). The full text was downloaded or collected from other sources. Articles of other languages were translated with the help of google translate and professionals if needed. The iBA-based AQUA was exploited to assess the study integrity or quality. Two of the authors read the full text and analyzed the merits based on the five domains of AQUA tools. The data of few studies pooled from literature reviews or other studies if the full text were not available were marked with unknown risk. We have found low to moderate risk and studies were suitable for data extraction for prevalence studies, but heterogeneity would be expected after combining the outcome. The reporting bias due to different methodologies could be possible because anatomical prevalence considered both cartilaginous and ossified fabella, but the radiological method only considered the ossified fabella. So the prevalence of fabella reported in anatomical studies or magnetic resonance imaging (MRI) might be higher as compared to radiographic prevalence due to existent cartilaginous fabella.

**Table 1:** Distribution of ossified fabella based on study characteristics, ethnicity, gender, and laterality. *Number fabella per 100 subjects.

Characteristics	No of studies	Prevalence	Lower bound	Upper bound
Methods				
Dissection	36	32%	26%	40%
Radiological	49	20%	16%	24%
Mode of study				
Anatomical	36	32%	26%	40%
CT scan	2	35%	19%	54%
MRI scan	7	28%	16%	43%
X-ray	39	19%	15%	24%
Risk of bias				
Low	66			
Moderate	14	22%	16%	30%
Unknown	6	19%	11%	31%
Ethnicity				
African	3	12%	6%	22%
Asian Caucasian	5	17%	11%	26%
Asian Mongoloid	32	41%	36%	47%
European	27	15%	12%	18%
North American	12	16%	12%	22%
Oceanian	3	48%	30%	66%
South American	4	18%	10%	30%
Gender				
Male	46	27%	22%	32%
Female	46	24%	18%	31%
Laterality*				
Bilateral	48	61%	26%	96%
Unilateral	48	27%	9%	46%

Data extraction and management 

The information of publishing year, population characteristics, mode of investigation (anatomical or radiological), sample size (number of individual or knee examined), and number of fabellae were extracted. The distribution of gender, age, laterality, and ethnicity were also noted. The contingency tables were prepared separately and the event rate with a 95% confidence interval was computed. Age, sex, and laterality were considered confounding factors, so the rate estimations were done separately. The unit of analysis for the prevalence of fabella was the number of fabella in 100 knees. The prevalence of fabella was measured with the help of ProMeta 3. The rate estimate for ossified fabella was measured with Revman 5.3 (The Nordic Cochrane Centre, The Cochrane Collaboration, Copenhagen, Denmark). The effect size and standard error for each study were computed with ProMeta 3. The heterogeneity statistic was determined as i^2^ statistics, Cochrane Q, and Kendall Tau. Random effect model was adopted if i^2^ was more than 50%, in place of the fixed-effect model. Q Cochrane was computed along with the P-value. Sensitivity and cumulative analysis were also performed. The cumulative meta-analysis was performed in order to evaluate the association prevalence of fabella with osteoarthritis and aging. The subgroup analysis was conducted to compute the rate according to ethnicity, mode of study, type of population, sex, and laterality. Regression analysis was carried out to examine the relationship between outcome and confounders (age, sex, and ethnicity). Finally, the odds ratio (OR), rate ratio (RR), and rate difference (RD) were computed. Funnel plots measured the publication bias. For the funnel plot, the logarithmically transformed odds ratio plotted against the standard error of each study. Additionally, the publication bias was measured with the help of Egger's linear regression test, Begg and Mazumdar's rank correlation test. Rosenthal Fail-Safe Number (FSN) was generated to refute the file drawer effect.

## Results

Description of studies 

**Figure 1:**
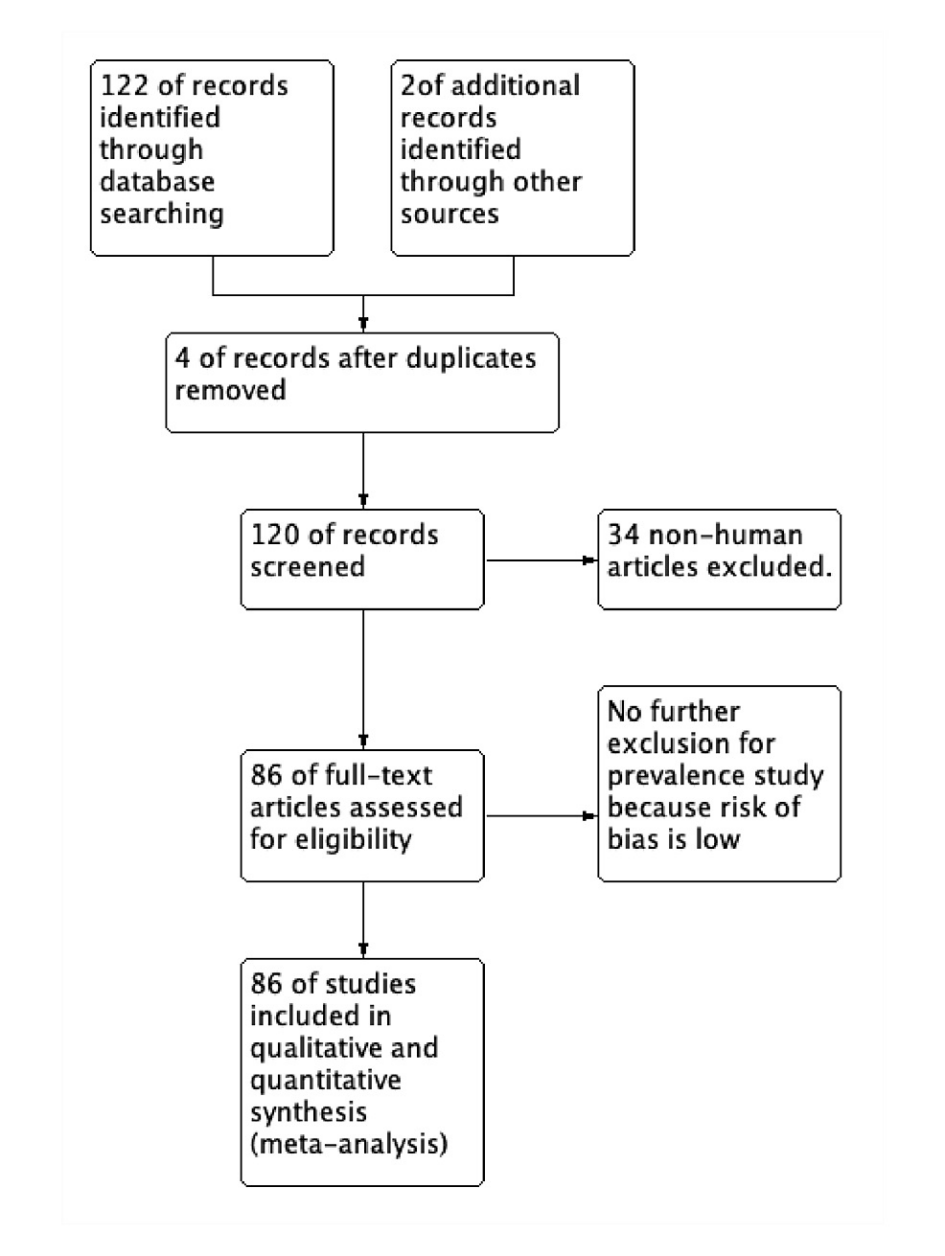
PRISMA flow diagram of the search strategy. PRISMA: Preferred Reporting Items for Systematic Reviews and Meta-Analyses.

The included publications were observational - cross-sectional, prospective, and retrospective studies. A total of 122 studies were identified by online research and two studies were found in conference proceedings. Due to duplicate titles or abstracts found during searches, four articles were eliminated. Thirty-six studies were excluded for being non-human studies or case reports and series, reviews-based abstract or title evaluation. A study was included after the consensus of both authors, and the third author was consulted in contradicting opinion. For the meta-analysis, 86 studies that dealt with the prevalence of fabella between 1875 and 2020 based on the abstract and full-text analysis were included in this study (Figure 1). The risk of bias was low to moderate. Fifty studies were radiological and 36 studies were cadaveric [1,3,5,7-43].

**Figure 2:**
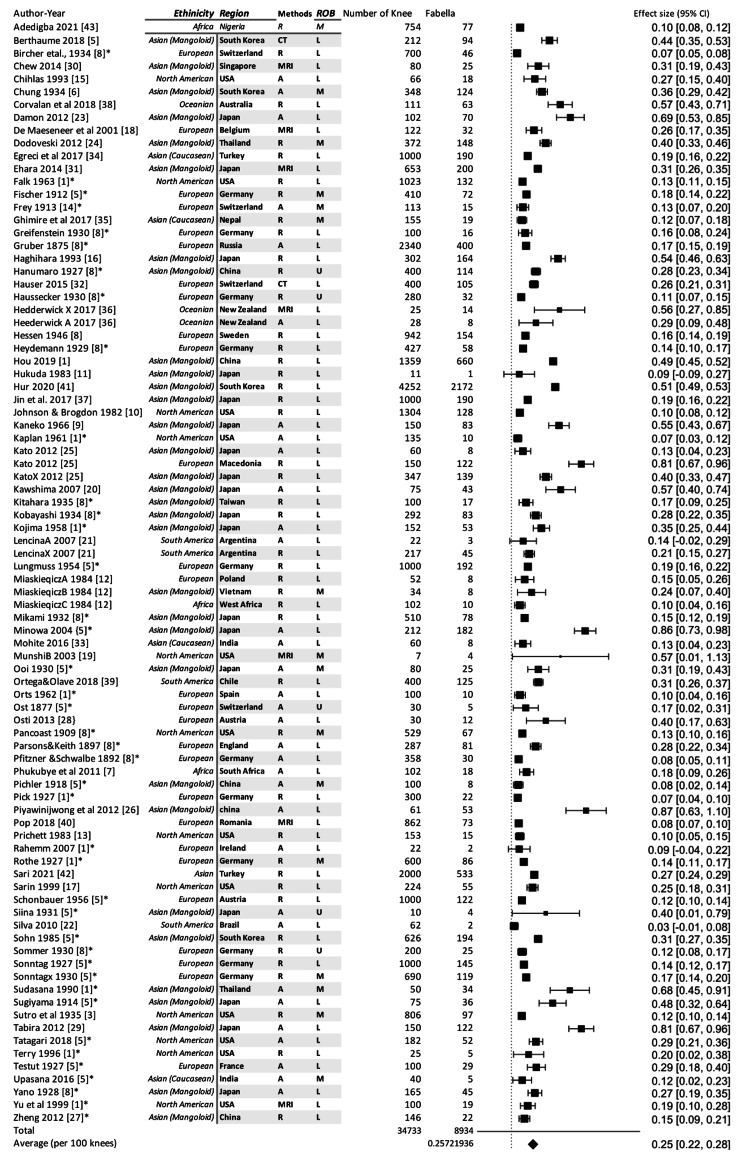
Forest plot computing the effective size of fabellar prevalence. A: anatomical studies, R: radiographic studies based on X-ray, ROB: risk of bias, L: low, M: moderate, U: unknown, H: high. *Secondary reference.

Totally, 34,733 knees were examined for the presence of fabella. The total number of reported fabellae was 8,934 and the prevalence of fabella was 25 per hundred knees (25% or 0.25) (Figure 2). Thirty-six cadaveric studies included 7,440 knees and the prevalence of fabellae was 32%. The FP in radiological studies was 20% on examination of 27,293 knees (Table 1). The existing difference between the two methods is because the cadaveric studies have counted both ossified and cartilaginous fabellae. The meta-regression was conducted to study the relationship between the publication year and FP which revealed that the FP was significantly increased from 1875 to 2020 (P=0.001) Then all studies were classified into 25 years interval for the period between 1875 and 2020 and again meta-analysis operation was conducted to delineate the rising prevalence. The fabellar prevalence was 17-19% for the duration of the years between 1875 and 1950, which got slightly increased to 23% during 1975-2000. A rapid increase to 34% FP was noted during 2000-2020 (almost double of FP of 1875-1950 as shown in Table 2).

**Table 2:** Prevalence of fabella at different time periods between 1875 and 2020. *Effect of two world wars. **Radiograph was invented in 1895 and the first medical X-ray was done in June 1896 by battlefield physicians to locate bullets in wounded soldiers. ^#^Prevalence of fabella (%)=0.568* life expectancy (in years) - 0.11, correlation coefficient = 0.56 (95% CI, 0.45-0.67), P<0.0001.

25 years interval	Studies (C+R)	Total prevalence (95%CI)	Cadaveric prevalence (95%CI)	Radiological prevalence (95%CI)
1875-1900	4+0	16% (8-30%)	17% (10-26%)	No studies**​​​​​​​
1901-1925	3+2	17% (10-30%)	20% (9-37%)	15% (6-32%)
1926-1950	5+15	18% (13-23%)	32%* (23-41%)	15% (12-18%)
1951-1975	4+3	19% (11-29%)	24% (14-26%)	15% (8-25%)
1976-2000	2+11	23% (16-32%)	27% (18-78%)	19% (11-31%)
2001-2020	18+19	35% (29-41%)	41% (31-52%)	28% (21-38%)
1875-2020^#^	36+50	25% (22-28%)	32% (26-40%)	20% (16-24%)

FP in Different Age-Group and Osteoarthritis

The FP in 0-10 years of the subject was 0.98% and it was increasing with age. The FP was 33.84% in more than 70 years of the aged population (Table 3). The rate of developing fabella was 1.19 at the age of 20 years which was increased to 1.81 by the age of 70 years. The mean age of the study population was showing a significant correlation with FP. Prevalence (%) =0.708* mean age of study population (in years) - 3.845, R=0.708, (95% CI, 0.568-0.809, P<0.001). The FP in OA knee was 51% but, the same in non-OA was 18%. The RR of developing fabella in the OA knee was 2.55. So, the mean age of the population and OA were determining FP.

**Table 3:** Distribution of fabella in different age groups and OA status of knees. ^#^The regression analysis of prevalence of fabella: prevalence (%)=0.708* age (in years) - 3.845, correlation coefficient=0.708, 95% CI (0.568-0.809), P<0.001. ^^^Overall rate ratio due to aging = 1.71(1.59-1.86).

Age^# ^(years)	Fabellar prevalence (%)	95% Confidence interval	Rate/risk ratio^ (95%CI)
0-10	0.98	0.74-1.26	NA (ossified at 8-12 years)
10-20	4.56	4.14-4.98	Baseline
20-30	14.52	13.77-15.27	1.19 (1.12-1.31)
30-40	18.88	18.02-19.73	1.5 (1.42-1.59)
40-50	23.44	22.49-24.39	1.63 (1.48-1.81)
50-60	25.38	24.39-26.36	1.62 (1.49-1.8)
60-70	29.45	28.39-30.51	1.64 (1.55-1.75)
>70	33.84	32.7-34.98	1.81 (1.86-2.21)
Non-OA	18	(7-28)	Baseline
OA	51	(45-57)	2.55 (2.15-3.02)

Ethnic and Geographical Distribution

**Figure 3:**
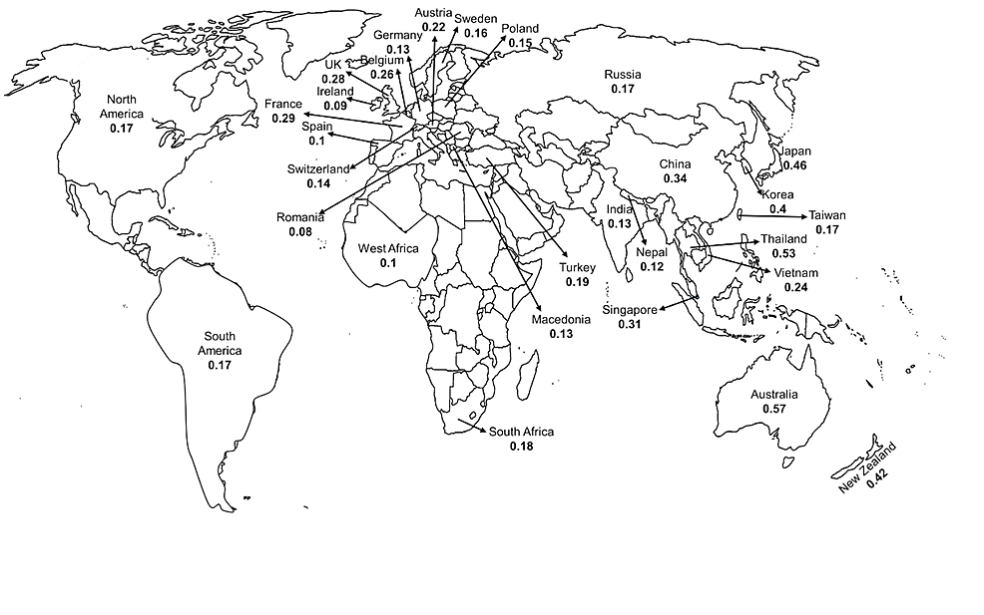
Geographical distribution of fabella (mean fabellar prevalence) observed during 1875-2020.

The prevalence of fabellae was very high in Oceanian populations (includes Australia and New Zealand; 48%) and Asian mongoloid populations (includes southeast Asian countries; 41%). The Oceanian population had the highest FP which would be an over-estimation with a wide confidence interval due to fewer studies (Table 1). The FP in Asian Caucasian, European, North, and South American population had almost similar confidence intervals (Figure 3). The African population had a slightly lower FP which would be due to the small sample size.

Sex and Laterality Distribution (Table 1)

FP was 27% in males and 24% in females. Bilateral occurrence of fabella was more common than unilateral occurrence. The laterality distribution was estimated per hundred subjects. The bilateral and unilateral occurrences were 61% and 27%, respectively. In the case of unilateral distribution, the right and the left side had equal distribution.

Methodological Distribution

The FP in dissected specimens was 32%. The FP in CT scan, MRI, and X-ray studies were 35%, 28%, and 19%, respectively (Table 1). Only two CT studies reported FP in which Hur et al. estimated the FP in OA of mongoloid population. So, the pooled FP for the CT scan was overestimated with a wider confidence interval. The FP in dissected specimens and MRI studies were almost similar in confidence intervals. The radiographic studies (X-ray) had lower FP because cartilaginous fabella was not observed and counted.

Publication bias

On examination of the funnel plot (asymmetrical funnel plot) and the result of Egger's regression test, some publication bias was suspected (P=0.02). But, Begg and Mazumdar's rank correlation test (P=0.126) refuted the asymmetrical funnel plot because of publication bias and it was possibly due to sample variation.

## Discussion

Fabellar presence is decided by the genetic constitution as seen in near-human species. The formation of fabella takes place in intrauterine life as a cartilaginous nodule. The cartilaginous nodule is derived from pre-cartilaginous condensations of fibroblast named as a sesamoid precursor in the gastrocnemius tendon during the embryonic period. Hox gene and genes encoding for the TGF-beta family played a crucial role in the sesamoid precursor. Drachman and Sokoloff studied the development of sesamoids in embryonic chicken. The above experiment had explained the fabella development not only determined by the genetic constitution but also by a biomechanical stimulus which led to epigenetic changes [44].

The present study demonstrated that the prevalence of fabella was 25% in 34,733 knees in over 150 years. The FP since the last two decades is estimated at 35% which was 16-18% in 1875-1950. The current meta-analysis has shown a doubling of FP. Berthaume and Bull [2] estimated that FP became 3.5 times in the last 150 years in 21,000 knees. They have computed a median FP which could be over-estimation due to lower sample size. According to them, the rising FP was either evolutionary or a certain biomechanical stimulus. They have tested the evolutionary model by taking the reference of Sarin et al. [17} but their evidence seems a hypothetical assumption without any scientific evidence. They presumed that an increased ratio of tibial and femoral (T/F) length and atrophy of gastrocnemius in the knee and hip osteoarthritis would be the stimulus for fabellar ossification [2]. Weinberg et al. documented that increased T/F ratio is a good predictor of hip and knee OA. They found that males, African and American races have higher T/F values than the female and white population [45]. If we presumed the above plea, then FP should be higher in Americans and Africans. But current data did not support this plea because Mongoloid and Oceanian population have higher fabellar prevalence than American or African populations. They have given another reason for this high prevalence in mongoloids. Kneeling, squatting, and tailor sitting are preferred by Mongoloids. Such habits may cause the fabella to strain against the posterior part of the lateral femoral condyle, promoting fabella formation, and ossification. But this plea is unable to explain the higher prevalence in Oceanian populations. The knee alignment significantly varied among different ethnic groups. The knee valgus alignment was highest for Oceanians, followed by Asian mongoloids, Caucasians, and Afro-Americans. The valgus alignment shifts the weight-bearing from the medial compartment to the lateral compartment. The FP was higher in lateral compartment OA [1,32]. Asian mongoloid like Chinese and Japanese have a valgus alignment of femoral angle in OA as compared to varus alignment seen in Caucasian, American, Middle-east, and Indian populations. Obesity and body mass index (BMI) may aggravate existing valgus knee alignment [46]. The loss of joint space and alteration of condyle plateau angle were attributed to such alignment [47,48]. This will shift the mechanical loading towards the lateral compartment which may lead to lateral tibiofemoral OA. Due to proximity with the femoral articular surface, the lateral tibiofemoral OA may induce osteoarthritic changes in fabella which would advance with age. Age-related degeneration and OA at the knee joint caused fabellar degeneration which resulted in fabellar ossification [1]. When the articular cartilage and menisci of the knee undergo OA changes, cartilaginous degeneration of fabella takes place, or sclerotic changes occur if fabella was already ossified. Following cartilaginous degeneration of fabella (fabellar degeneration), it ossifies or gets sclerosed, leading to fabellar enlargement [1]. The earlier meta-analysis has demonstrated a significant association of FP with OA which could be explained by fabellar degeneration [49]. The possible mechanism is the calcification of articular cartilage, menisci, and fabellar cartilage following degeneration. OA might be the reason behind the increased FP as its prevalence has doubled from 1950 to 2000. The prevalence of OA has increased because of improved life expectancy worldwide [49,50].

**Figure 4:**
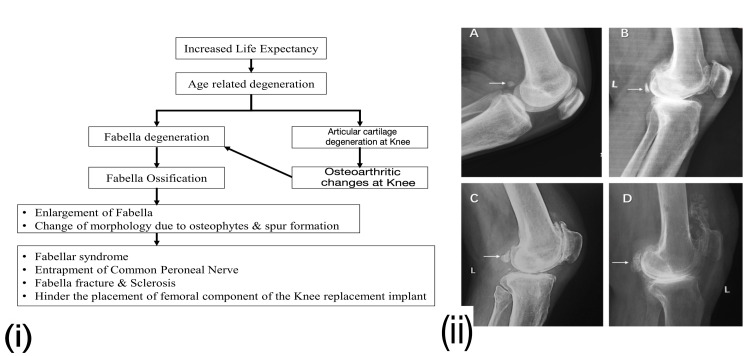
(i) Fabellar degeneration and ossification flow diagram. (ii) Different grades of fabellar degeneration and ossification are shown in the lateral knee radiographs. (A) Normal fabella: oval-shaped with anterior smooth surface articulating with the posterolateral condyle of the femur (arrow); (B) sclerosed fabella: subchondral sclerosis of anterior surface (arrow); (C) severe sclerosis of the fabella with osteophyte formation (arrow); (D) enlargement of fabella with marked osteophyte formation (arrow) [1]. Permitted under Creative Commons Attribution 4.0.

Fabella is exposed to such changes due to its vicinity to the knee joint. Such changes contribute to alteration in the shape and size of fabella (Figure 4). The enlarged fabella may cause compression of surrounding structures like nerve or tendon or implants. Knee valgus alignment and obesity are also possible contributors [48].

Limitation of study

Despite the fact that this study offers useful information about the prevalence of fabella, and its relation with OA, the main limitation of this study is the inadequacy of information regarding weight, height, and BMI. The studies used in the assessment were of various ethnicities and methodologies, leading to even greater heterogeneity. Height, gender, physical habitus or profession, and BMI all seem to be potential confounders in the pooled estimates. Prevalence estimates can be influenced by sample variance.

## Conclusions

This study demonstrated the doubling of FP in the last 70 years. OA knee has three times FP of baseline. The incidence of fabella is higher in the Oceanian and Mongoloid populations, male participants, and on the right side of the knee, which is consistent with previous findings. Bilateral occurrence of fabella is far more common than unilateral occurrence, which corresponds to OA distribution. The valgus positioning of the knee, along with obesity, causes lateral compartment OA in the knee, which can raise the FP. The further scope of the study is to evaluate the prevalence of fabella in severe osteoarthritis with and without valgus knee alignment. The stratification of the proposed study would include a final estimation of FP based on the biomechanical load on the knee, as well as aid in understanding the function of fabella in knee biomechanics.

## Appendices

Prospero registration: CRD42020161834 (dated 28-04-2020)

Search strategy (PubMed):

(((((((Fabella[Title/Abstract]) OR Knee sesamoid[Title/Abstract]) OR Popliteal Sesamoid[Title/Abstract]) OR Sesamoid[Title/Abstract])) AND ((((Prevalence[Title/Abstract]) OR Incidence[Title/Abstract]) OR event rate[Title/Abstract]) OR events[Title/Abstract])) AND (((((Osteoarthritis[Title/Abstract]) OR Knee Degeneration[Title/Abstract]) OR Knee Pain[Title/Abstract]) OR Knee aging[Title/Abstract]) OR Genu pain[Title/Abstract])) NOT Animal').
